# Development of an evaluation method for addictive compounds based on electrical activity of human iPS cell‐derived dopaminergic neurons using microelectrode array

**DOI:** 10.1111/adb.13443

**Published:** 2024-10-09

**Authors:** Yuto Ishibashi, Nami Nagafuku, Shingo Kimura, Xiaobo Han, Ikuro Suzuki

**Affiliations:** ^1^ Department of Electronics, Graduate School of Engineering Tohoku Institute of Technology Sendai Miyagi Japan

**Keywords:** drug addiction, human iPS cell‐derived dopaminergic neurons, in vitro, microelectrode array, new approach methodologies, principal component analysis

## Abstract

Addiction is known to occur through the consumption of substances such as pharmaceuticals, illicit drugs, food, alcohol and tobacco. These addictions can be viewed as drug addiction, resulting from the ingestion of chemical substances contained in them. Multiple neural networks, including the reward system, anti‐reward/stress system and central immune system in the brain, are believed to be involved in the onset of drug addiction. Although various compound evaluations using microelectrode array (MEA) as an in vitro testing methods to evaluate neural activities have been conducted, methods for assessing addiction have not been established. In this study, we aimed to develop an in vitro method for assessing the addiction of compounds, as an alternative to animal experiments, using human iPS cell‐derived dopaminergic neurons with MEA measurements. MEA data before and after chronic exposure revealed specific changes in addictive compounds compared to non‐addictive compounds, demonstrating the ability to estimate addiction of compound. Additionally, conducting gene expression analysis on cultured samples after the tests revealed changes in the expression levels of various receptors (nicotine, dopamine and GABA) due to chronic administration of addictive compounds, suggesting the potential interpretation of these expression changes as addiction‐like responses in MEA measurements. The addiction assessment method using MEA measurements in human iPS cell‐derived dopaminergic neurons conducted in this study proves effective in evaluating addiction of compounds on human neural networks.

## Graphical abstract

Microelectrode array (MEA) data before and after chronic exposure revealed specific changes in addictive compounds compared to non‐addictive compounds, demonstrating the ability to estimate addiction of compound. The addiction assessment method using MEA measurements in human iPS cell‐derived dopaminergic neurons conducted in this study proves effective in evaluating addiction of compounds on human neural networks.

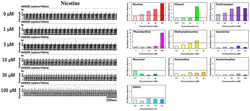

## 1 INTRODUCTION

Drug addiction is characterized by a chronic and recurrent condition in which a strong desire to consume a drug arises due to the euphoria and pleasure obtained from drug use, despite the presence of side effects.^1^ Multiple neural networks, including the reward system, anti‐reward/stress system and central immune system in the brain, are believed to be involved in the onset of drug addiction.^2^ Specifically, the relationship between addictive compounds and dopaminergic neurons has been extensively reported. For example, nicotine, found in tobacco, has been reported to alter synaptic plasticity and affect dopamine signalling.^3^ Ethanol, a type of alcohol, has been reported to change the activity of dopaminergic neurons with chronic consumption.^4^ Flunitrazepam, a sleep aid, is reported to influence dopamine release from the striatum.^5^ Phenobarbital has been reported to alter dopamine receptor characteristics when exposed to mice at an early developmental stage.^6^ Methamphetamine has been reported to cause sustained dopamine depletion with repeated use.^7^

Clinically, drug addiction is diagnosed through standardized assessments such as the Diagnostic and Statistical Manual of Mental Disorders, Fifth Edition (DSM‐V), in the United States. In non‐clinical settings, it is believed that the evaluation of the psychotropic effects of drugs can be assessed by measuring drug‐taking behaviour and reward effects in animals.^8^ The most reliable method for assessing the psychotropic effects of drugs is considered to be drug self‐administration.^9^, ^10^ Additionally, conditioned place preference tests are widely used as a relatively convenient method to assess the rewarding effects of drugs.^11^, ^12^, ^13^ However, in animal experiments that require substantial time and cost, there is no established method for the evaluation of addiction on novel compounds, which may be highly useful for the rapid assessment of newly emerging illicit drugs or the screening of a diverse range of compounds for potential drug candidates, both of which demand prompt evaluation.^2^, ^14^

As an example of efforts to assess the impact of chemical substances on neural networks, there is a development of assessment methods for developmental neurotoxicity (DNT), and a strategy is anticipated for the development of in vitro testing methods utilizing new approach methodologies.^15^, ^16^ The development of in vitro testing methods for chemical‐induced neurotoxicity, including DNT, faces challenges due to the complex nature and diverse functions of the nervous system. Capturing the mechanisms of neurotoxicity induced by various chemical substances is difficult, and currently, there is no established in vitro testing method that can replace in vivo neurotoxicity evaluation.^17^, ^18^, ^19^ Recently, guidance on in vitro testing for developmental neurotoxicity has been discussed as an OECD guideline for chemical testing, and the concept of an in vitro testing battery combining multiple in vitro tests has been introduced. In the field of addiction assessment, the establishment of high‐throughput in vitro testing methods could potentially be utilized for the regulation of illicit drugs and the screening of potential drug candidates.

In vitro testing methods to evaluate neural activities include patch clamp,^20^, ^21^ calcium imaging^22^, ^23^ and extracellular recording using microelectrode array (MEA). Among measurable assessment systems for in vitro neural function, MEA allows non‐invasive, high temporal resolution and simultaneous multi‐point recording of the electrophysiological activity of neural networks. Due to its ability to accurately and high‐throughput evaluate changes in neural network activity caused by compound administration, MEA is expected to serve as an in vitro neurofunctional indicator for chemical substance assessment, replacing animal experiments.^24^, ^25^, ^26^, ^27^ Studies employing MEA measurements with rodent neural cells have reported its potential as a valuable assessment system to identify compounds inducing seizure‐like activity.^28^, ^29^, ^30^ MEA measurements using human‐induced pluripotent stem (iPS) cell‐derived neurons have also reported evaluations of neural network activity, including spontaneous activity and responses to seizurogenic compounds.^31^, ^32^, ^33^ Furthermore, recent research utilizing MEA has introduced high‐resolution measurement methods, such as brain organoid activity monitoring and CMOS‐MEA with high‐density electrodes, elevating expectations for MEA.^34^, ^35^, ^36^ Research on addiction using MEA has reported the development of brain organoids that form functional long‐distance dopaminergic connections, which are expected to serve as useful in vitro models for addiction assessment.^37^ Although various compound evaluations using MEA and neural cells have been conducted, methods for assessing addiction have not been established, and the development of chronic administration test methods that reproduce the onset of drug addiction remains a challenge.

In this study, we aimed to develop an in vitro method for assessing the addiction of compounds, as an alternative to animal experiments, using human iPS cell‐derived dopaminergic neurons with MEA measurements. To evaluate the addiction of compounds, human iPS dopaminergic neurons were chronically exposed to the compounds, and the impact on neural activity of both addictive and non‐addictive compounds was assessed through MEA measurements before and after chronic exposure. Principal component analysis (PCA) of electrophysiological parameters calculated from MEA data before and after chronic exposure revealed specific changes in addictive compounds compared to non‐addictive compounds, demonstrating the ability to estimate addiction of compound. The addiction assessment method using MEA measurements in human iPS cell‐derived dopaminergic neurons conducted in this study proves effective in evaluating addiction of compounds on human neural networks. This method holds promise as an in vitro addiction testing approach, serving as an alternative to animal experiments.

## 2 MATERIALS AND METHODS

### 2.1 Culture of human iPSC‐derived neurons

Human iPS cell‐derived dopaminergic neurons (iCell DopaNeurons 01279, FUJIFILM Cellular Dynamics, Inc.) were seeded onto a 24‐well MEA plate (M384‐tMEA‐24 W, Axion BioSystems, Inc.). The MEA plate was coated with 0.07% polyethyleneimine (FUJIFILM Wako) the day before seeding. The culture medium used was a mixture of Complete Brainphys Medium (comprising 95 ml BrainPhys Neuronal Medium [STEMCELL Technologies], 2 ml iCell Neural Supplement B [FUJIFILM Cellular Dynamics, Inc.], 1 ml iCell Neuro System Supplement [FUJIFILM Cellular Dynamics, Inc.], 0.1 ml Laminin [Sigma‐Aldrich], 1 ml N‐2 Supplement [×100, Gibco] and 1 ml Penicillin/Streptomycin [FUJIFILM wako]) and Laminin, which was diluted 100 times (referred to as Complete Brainphys Medium+ Laminin). The plate was coated with iMatrix511 (Matrixome Inc.) and incubated at 37°C with 5% CO2 for at least 1 h. After removing iMatrix511 from the plate, cells were seeded at a density of 7.3 × 10^4^ cells per well using a cloning ring. The plate was then incubated at 37°C with 5% CO2 for 60 min to allow cell attachment to the bottom of the MEA. Once cell attachment was confirmed by microscopic observation, the cloning ring was removed, and each well was supplemented with 700 μl of Complete Brainphys Medium+ Laminin. The cells were cultured in the incubator. Half of the culture medium was replaced with fresh Complete Brainphys Medium on Days 1, 2, 5 and 7 after seeding.

### 2.2 Culture of human iPSC‐derived astrocytes

Human iPS cell‐derived astrocytes (iCell Astrocytes 01434, FUJIFILM Cellular Dynamics, Inc.) were seeded onto a six‐well glass plate. The wells for cultivation were coated with Geltrex (Gibco) before seeding the astrocytes. The culture medium used was iCell Astrocyte Medium (comprising 89 ml DMEM (high glucose, GlutaMAX, pyruvate), 10 ml FBS, 1 ml N‐2 Supplement (×100), FUJIFILM Cellular Dynamics, Inc.). On the day following seeding, the entire medium was exchanged, and subsequent complete medium exchanges were performed at 2–3 day intervals.

### 2.3 Co‐culture of human iPSC‐derived neuron and astrocytes

On the 7th day of dopaminergic neuron culture, astrocytes previously cultured were added at a cell density of 2.0 × 10^4^ cells. Using Complete Brainphys Medium, half of the medium was exchanged every 2–3 days until 21 days after seeding dopaminergic neurons. After 21 days of seeding dopaminergic neurons, the medium was changed from Complete Brainphys Medium to Complete Maturation Medium (485 ml BrainPhys Neuronal Medium, 10 ml NeuroCult SM1 Neuronal Supplement, 5 ml Penicillin/Streptomycin), and subsequently, half of the medium was exchanged every 2–3 days.

### 2.4 Extracellular recordings

Spontaneous extracellular field potentials were acquired at 37°C and 5% CO_2_ using the 24‐well MEA system (Maestro Pro, Axion BioSystems, Inc.) at a sampling rate of 12.5 kHz per channel. Spikes were detected in the acquired data using a 200 Hz high‐pass filter.

### 2.5 Pharmacological tests

**FIGURE 1:**
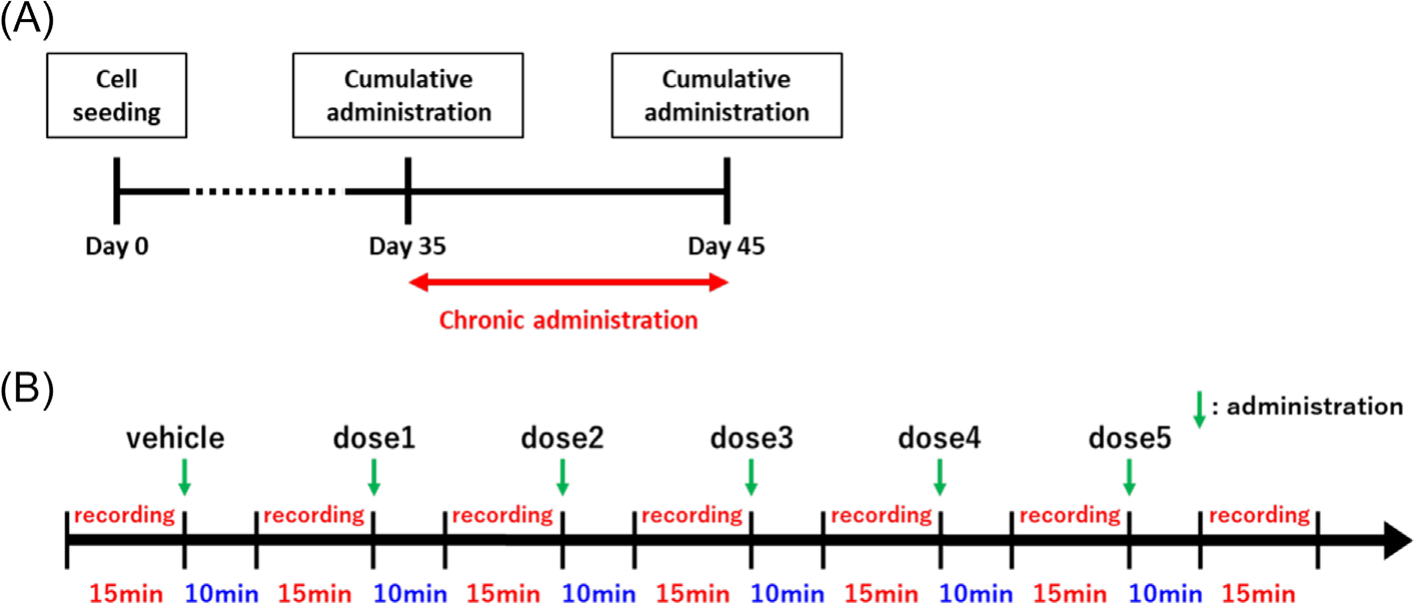
Schematic timeline for the experimental design. (A) Timeline of the whole experiment, including the chronic administration period. (B) Timeline of the cumulative administration experiment.

Pharmacological tests were performed in human iPSC‐derived neurons after culturing for 5 weeks, when sufficient spikes were detected. Table 1 shows details on the test compounds. The final concentration of these drugs was adjusted to 0.1% DMSO, which was added to all wells as vehicle control prior to cumulative dosing with the compounds. Figure 1 shows schematic timeline for the experimental design. Spontaneous firing was recorded for 15 min at each concentration (*n* ≥ 8). To reduce the effects of desensitization, a standing time of 10 min was provided between recordings. Pharmacological tests for each drug were conducted on 35 DIV (before chronic administration) and 45 DIV (after chronic administration), and from 35 DIV to 45 DIV, culture was continued in a medium mixed with the drug to establish chronic administration.

**TABLE 1:** Details of test compounds.

Compound	Class	Cumulative (μM)	Chronic (μM)	Source	#CAS
Nicotine	nAChRs agonist (addictive)	1, 3, 10, 30, 100	1	Sigma‐Aldrich	54‐11‐5
Ethanol	GABA_A_ R agonist (addictive)	0.03%, 0.1%, 0.3%, 1%, 3%	0.03%	Sigma‐Aldrich	64‐17‐5
Flunitrazepam	GABA_A_ R agonist (addictive)	0.1, 0.3, 1, 3, 10	0.1	Sigma‐Aldrich	1622‐62‐4
Phenobarbital	GABA_A_ R agonist (addictive)	1, 3, 10, 30, 100	1	Sigma‐Aldrich	50‐06‐6
Methamphetamine	TAAR1 agonist (addictive)	0.3, 1, 3, 10, 30	0.3	Sumitomo Pharma	537‐46‐2
Varenicline	nAChRs agonist (non‐addictive)	0.1, 0.3, 1, 3, 10	0.1	Sigma‐Aldrich	375815‐87‐5
Muscimol	GABA_A_ R agonist (non‐addictive)	3, 10, 30, 100, 300, 1,000	3	Sigma‐Aldrich	2763‐96‐4
Amantadine	Dopamine agonist (non‐addictive)	0.03, 0.1, 0.3, 1, 3	0.03	Sigma‐Aldrich	665‐66‐7
Acetaminophen	Negative control (non‐addictive)	1, 3, 10, 30, 100	1	Sigma‐Aldrich	103‐90‐2
DMSO	Vehicle	0.2%, 0.3%, 0.4%, 0.5%, 0.6%	0.2%	Sigma‐Aldrich	67‐68‐5

*Note*: Details of five addictive compounds and five non‐addictive compounds.

### 2.6 Spike detection and burst analysis

Electrophysiological activity was examined using the AxIS navigator software (Axion Inc.) and MATLAB. A spike was considered when the extracellularly recorded signal was >±5.3 σ, where σ is the standard deviation (SD) of the baseline noise during quiescent periods. Network bursts (NBs) were detected using a four‐step method.^38^ Table 2 shows description of analytical parameters. The response to compound administration was evaluated using 13 analytical parameters: total spikes (TSs), number of NBs, inter‐burst interval (IBI), duration of NB (duration), spikes in an NB (spikes), maximum frequency (MF), inter‐MF interval (IMFI), coefficient of variation of duration (CV of Duration), coefficient of variation of spikes (CV of Spikes), coefficient of variation of MF (CV of MF), coefficient of variation of IMFI (CV of IMFI), periodicity and duration IQR. MF was calculated by counting the number of spikes in every 100‐ms bin size from each NB detected in 15 min and averaging the maximum number of spikes in each. For example, if 10 NBs were detected in 15 min, the maximum number of spikes for each of the 10 NBs was calculated and averaged to obtain the MF. The normalization of pharmacological test data was performed by assuming that vehicle administration in each well was 100%. TS, number of NBs, IBI, duration and spikes were calculated using the four‐step method, whereas MF, IMFI, CV of duration, CV of spikes, CV of MF, CV of IMFI, periodicity and duration IQR were evaluated using MATLAB.

**TABLE 2:** Description of analytical parameters.

Analytical parameter	Description
Total spikes (TS)	The total number of spikes detected on all channels in 15 min
No. of network bursts (No. of NBs)	The number of NBs in 15 min
Inter‐burst interval (IBI)	Average time from the end point of NB to the start point of the next NB
Duration of network burst (Duration)	Average duration of NBs
Spikes in a network burst (Spikes)	Average the number of spikes contained in an NB
Max frequency (MF)	Average peak value of the histogram during an NB
Inter‐MF interval (IMFI)	Average time from the peak of NB to the peak of the next NB
CV of Duration	Coefficient of variance of duration
CV of Spikes	Coefficient of variance of spikes
CV of MF	Coefficient of variance of MF
CV of IMFI	Coefficient of variance of IMFI
Periodicity	The periodicity of the neural network activity in measurement time of all
Duration IQR	Interquartile range of network burst duration

*Note*: A list and description of the 13 analytical parameters calculated by burst analysis.

### 2.7 Statistical analysis

One‐way ANOVA followed by Dunnett's test were used to calculate significant differences between each compound and vehicle in burst analysis (*p* < 0.05).

### 2.8 Principal component analysis

A matrix was constructed using the 13 analytical parameters obtained by the burst analysis of test compounds at each concentration. PCA was performed using the MATLAB function PCA on 8178 parameter sets to select ≥2 parameters among the 13 analytical parameters. One‐way MANOVA was used to calculate significant differences between each concentration of the first two principal components. To detect specific changes in addictive compounds before and after chronic administration, we explored parameter sets where no significant differences were observed before and after chronic administration in non‐addictive compounds, while significant differences were observed only in addictive compounds. If multiple parameter sets were extracted, the parameter set with the smallest *p* value was identified and designated as the most significant parameter set. Using the identified parameter set and principal component loadings, PCA was performed on five addictive compounds and five non‐addictive compounds. Furthermore, to quantify the changes in neuronal activity responses due to chronic administration of the compounds, the distance between the plots before and after chronic administration of the same compound at the same concentration was calculated on a 2D plane of the PCA. The threshold was set at 2SD of the distance before and after chronic administration of DMSO, with changes exceeding this threshold being evaluated as addiction‐like responses and changes not exceeding this threshold being evaluated as non‐addiction responses.

### 2.9 Genomics library preparation and sequencing analysis

RNA from culture samples was extracted immediately after testing on Day 45. After MEA measurements, neurons were lysed directly in the culture well by addition of 500 μl TRIzol™ Reagent (15596026, Thermo Fisher Scientific). Total RNA was extracted manually using chloroform and isopropyl alcohol solution following manufacturers protocol. An RNA sequencing analysis was entrusted to Agenta Co., Ltd, Tokyo. Briefly, mRNA was extracted by the poly‐A selection method targeting mRNA, and the whole genome sequencing was performed using HiSeq X Ten (Illumina Inc.). Expression levels of all mRNAs were presented by the calculated value of fragments per kilobase of exon per million reads mapped (FPKM). The whole FPKM value of neuron samples under three conditions (after long‐term exposure to DMSO, nicotine or varenicline, *n* = 1 sample each) was analysed, and the expression levels of several typical mRNAs were manually picked‐up.

## 3 RESULTS

### 3.1 Measurement of pharmacological responses in human iPS cell‐derived dopaminergic neurons

**FIGURE 2:**
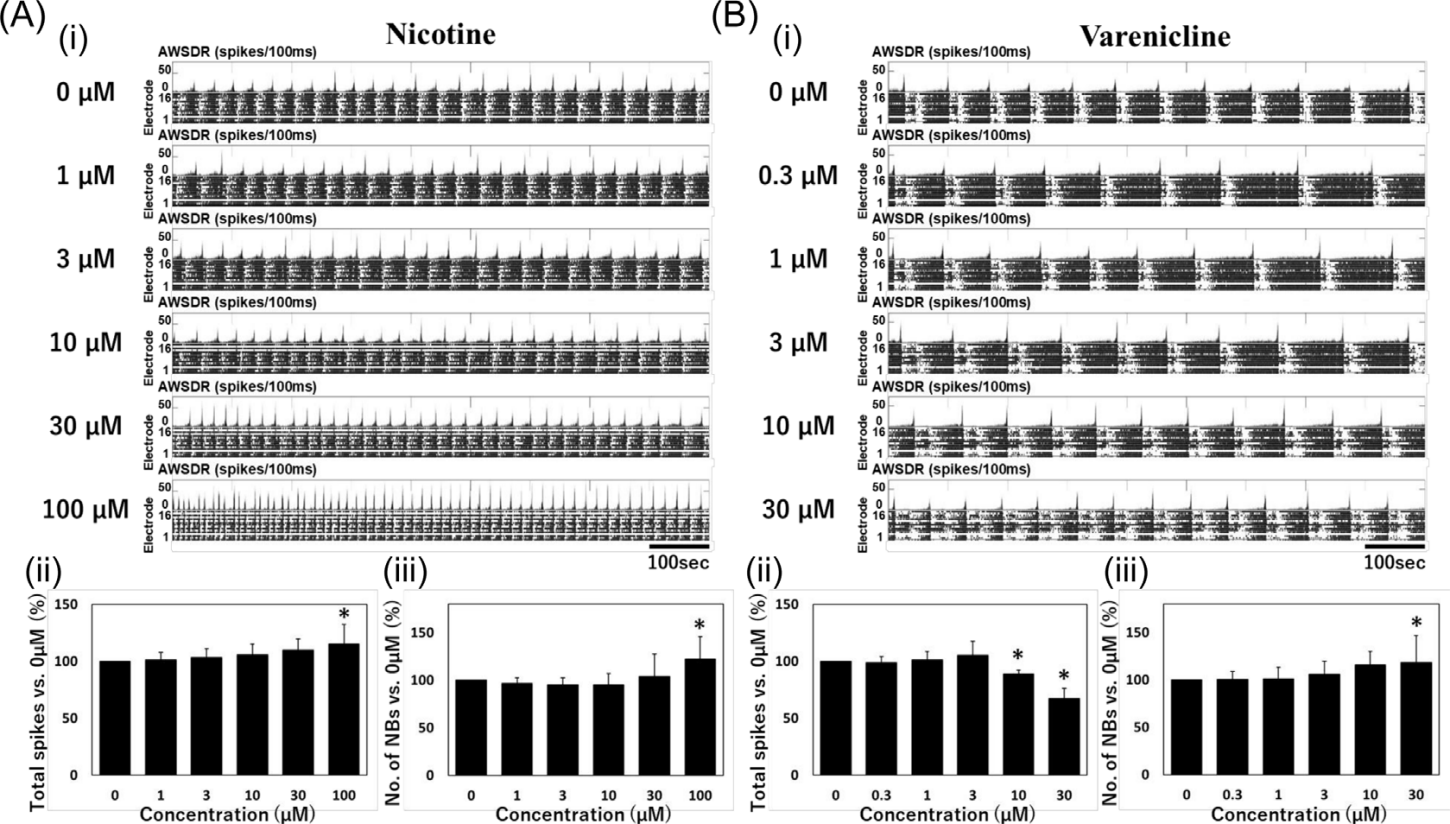
Electrophysiological responses of human iPS cell‐derived dopaminergic neurons to nicotine and varenicline. (A) Electrophysiological responses to nicotine (*n* = 10 well). (i) Raster plot (upper) and histogram (lower). (ii) Total spikes. (iii) Number of network bursts. (B) Electrophysiological responses to varenicline (*n* = 10 well). (i) Raster plot and histogram. (ii) Total spikes. (iii) Number of network bursts. Error bars indicate the standard deviation. One‐way ANOVA followed by Dunnett's test, *p < 0.05 versus 0 μM. AWSDR, array wide spike detection rate.

Electrophysiological responses of human‐induced pluripotent stem cell‐derived dopamine neurons on DIV 35 (before chronic administration) to nicotine and varenicline are demonstrated (Figure 2). Concentration‐dependent responses to nicotine and varenicline were observed from raster plots and histograms. Nicotine showed an increase in concentration‐dependent firing rate and number of NBs (Figure 2A). On the other hand, varenicline exhibited a concentration‐dependent decrease in firing rates and an increase in number of NBs (Figure 2B).

### 3.2 Electrophysiological responses of human iPS cell‐derived dopaminergic neurons before and after chronic administration

**FIGURE 3:**
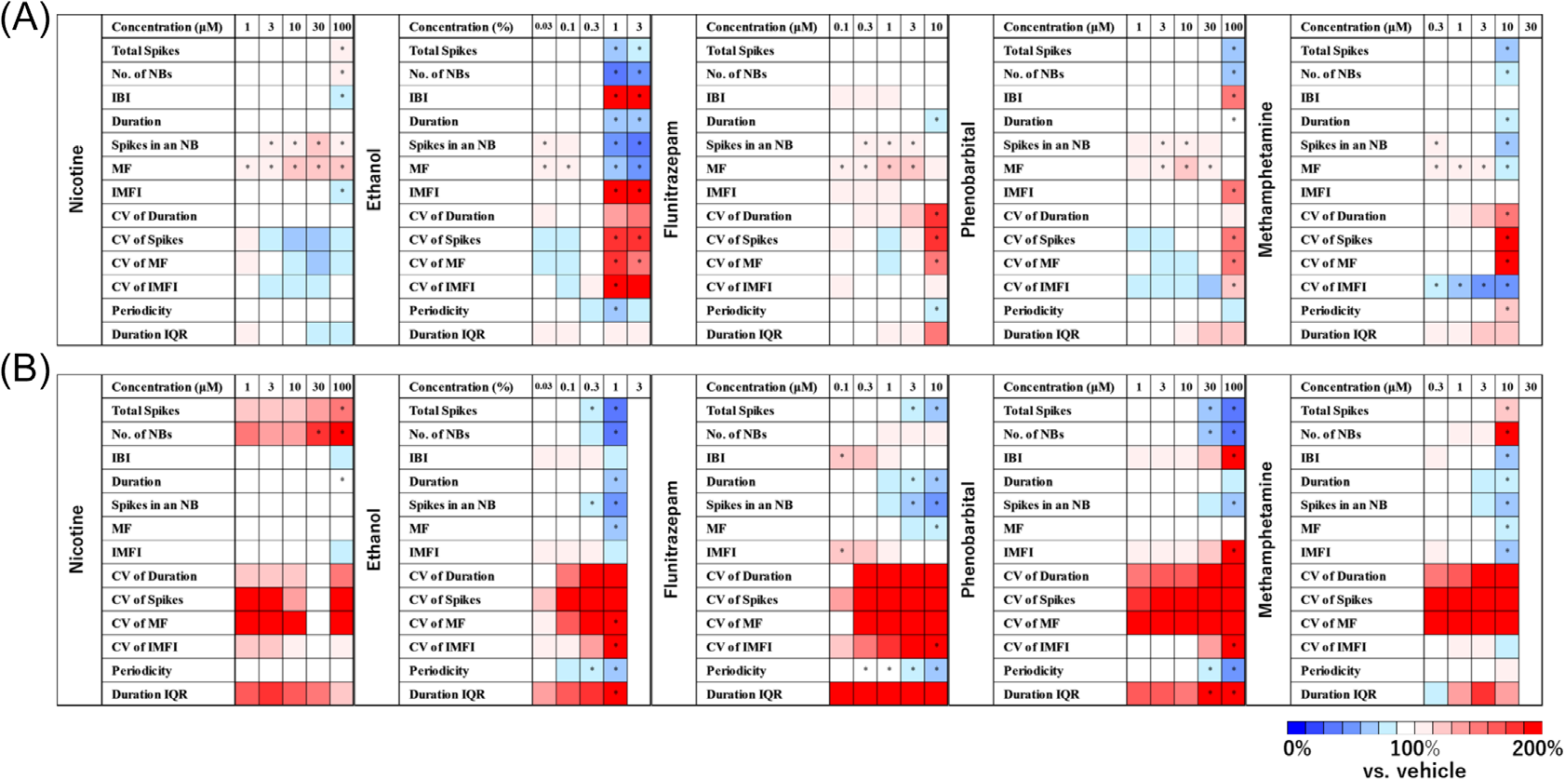
Electrophysiological responses of human iPS cell‐derived dopaminergic neurons to addictive compounds before and after chronic administration. (A) Heatmap of electrophysiological parameters for addictive compounds before chronic administration. Nicotine (*n* = 10 well), ethanol (*n* = 10 well), flunitrazepam (*n* = 10 well), phenobarbital (*n* = 10 well) and methamphetamine (*n* = 10 well). (B) Heatmap of electrophysiological parameters for addictive compounds after chronic administration. Nicotine (*n* = 10 well), ethanol (*n* = 10 well), flunitrazepam (*n* = 10 well), phenobarbital (*n* = 10 well) and methamphetamine (*n* = 10 well). One‐way ANOVA followed by Dunnett's test, *p < 0.05 versus vehicle.

**FIGURE 4:**
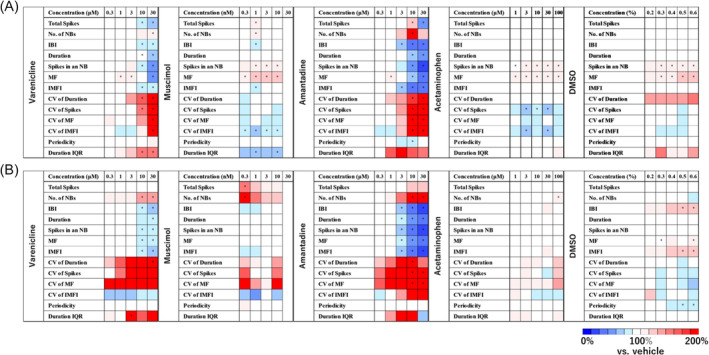
Electrophysiological responses of human iPS cell‐derived dopaminergic neurons to non‐addictive compounds before and after chronic administration. (A) Heatmap of electrophysiological parameters for non‐addictive compounds before chronic administration. Varenicline (*n* = 10 well), muscimol (*n* = 8 well), amantadine (*n* = 8 well), acetaminophen (*n* = 10 well) and DMSO (*n* = 10 well). (B) Heatmap of electrophysiological parameters for non‐addictive compounds after chronic administration. Varenicline (*n* = 10 well), muscimol (*n* = 8 well), amantadine (*n* = 8 well), acetaminophen (*n* = 10 well) and DMSO (*n* = 10 well). One‐way ANOVA followed by Dunnett's test, *p < 0.05 versus vehicle.

For the cumulative dosing experiments of each drug before and after chronic administration, 13 electrophysiological parameters (TSs, number of NBs, IBI, NB interval, duration, spikes in an NB, MF, IMFI, CV of duration, CV of spikes, CV of MF, CV of IMFI, periodicity and duration IQR) were calculated, and a heatmap was generated. Changes in different parameters before and after chronic administration were observed with addictive compounds. For nicotine, an increase in TSs and number of NBs was highlighted after chronic administration, while increases in spikes in an NB and MF were no longer observed. For ethanol, a decrease in TSs and number of NBs was highlighted after chronic administration, and NBs disappeared at the highest concentration. For flunitrazepam, spikes in an NB and MF decreased after chronic administration. For phenobarbital, a decrease in TSs and number of NBs was emphasized after chronic administration. For methamphetamine, TSs and number of NBs increased after chronic administration. Additionally, an increase in the CV parameter was observed after chronic administration for all addictive compounds, indicating a loss of regularity in firing within NBs. (Figure 3). However, changes in different parameters before and after chronic administration were also observed with non‐addictive compounds. For varenicline, the decrease in TSs disappeared after chronic administration, and the increase in number of NBs was highlighted. For muscimol, the increase in TSs and number of NBs was emphasized after chronic administration, while the increases in spikes in an NB and MF were no longer observed. An increase in the CV parameter was also observed after chronic administration for non‐addictive compounds, indicating the need to identify parameters that can detect the characteristic responses of addictive compounds. (Figure 4).

### 3.3 Detection of addictive compounds through PCA

**FIGURE 5:**
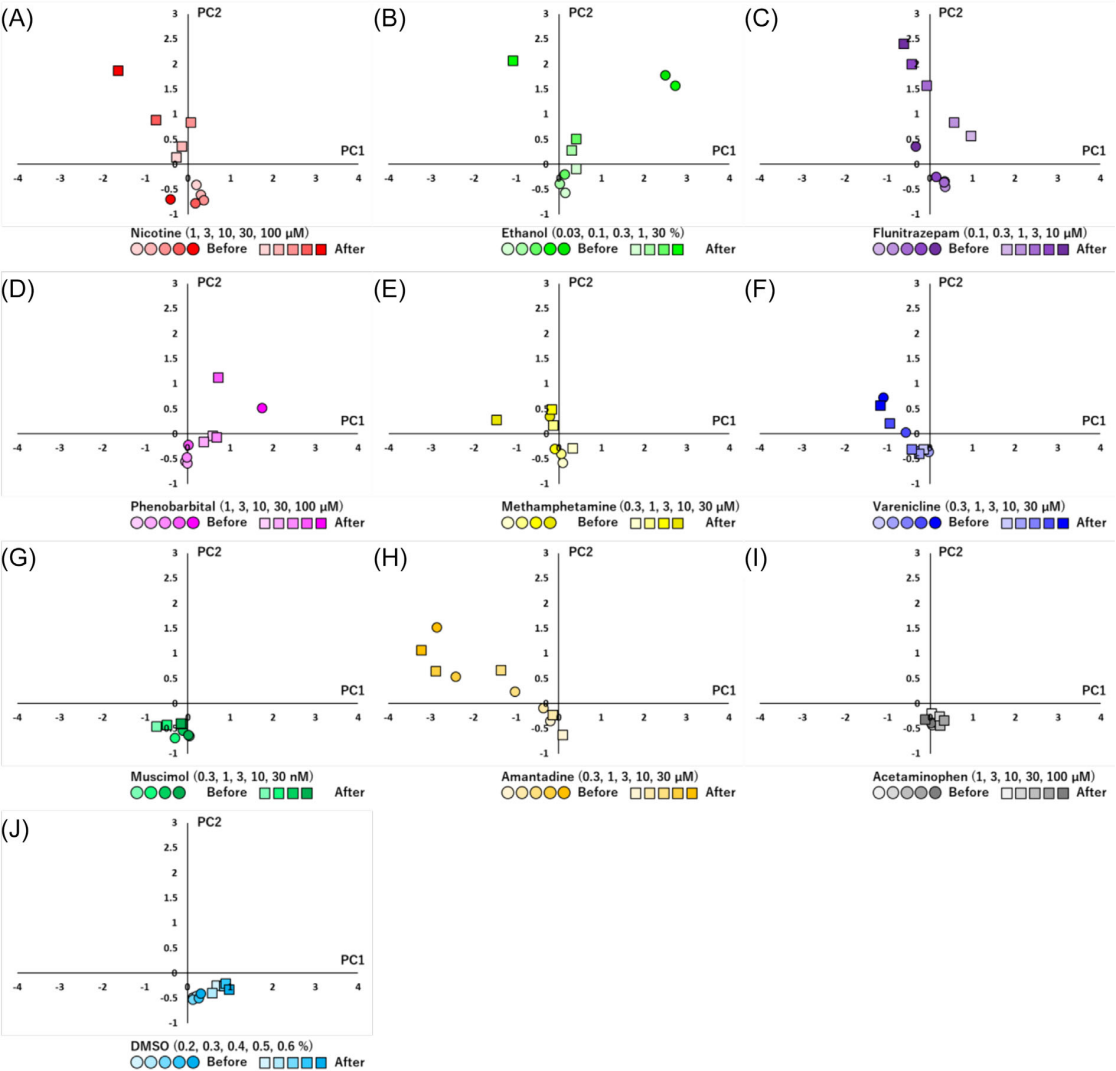
Comparative analysis of responsiveness before and after chronic administration for addictive and non‐addictive compounds using PCA. PCA using effective analytical parameters to detect differences before and after chronic administration of addictive compounds. Plots per data point indicate the centroids. Before chronic administration (circle), after chronic administration (square). (A) Nicotine (*n* = 10 well), (B) ethanol (*n* = 10 well), (C) flunitrazepam (*n* = 10 well), (D) phenobarbital (*n* = 10 well), (E) methamphetamine (*n* = 10 well), (F) varenicline (*n* = 10 well), (G) muscimol (*n* = 8 well), (H) amantadine (*n* = 8 well), (I) acetaminophen (*n* = 10 well) and (J) DMSO (*n* = 10 well). Contribution rate of the PC1 is 52.4%, and PC2 is 29.2%.

**FIGURE 6:**
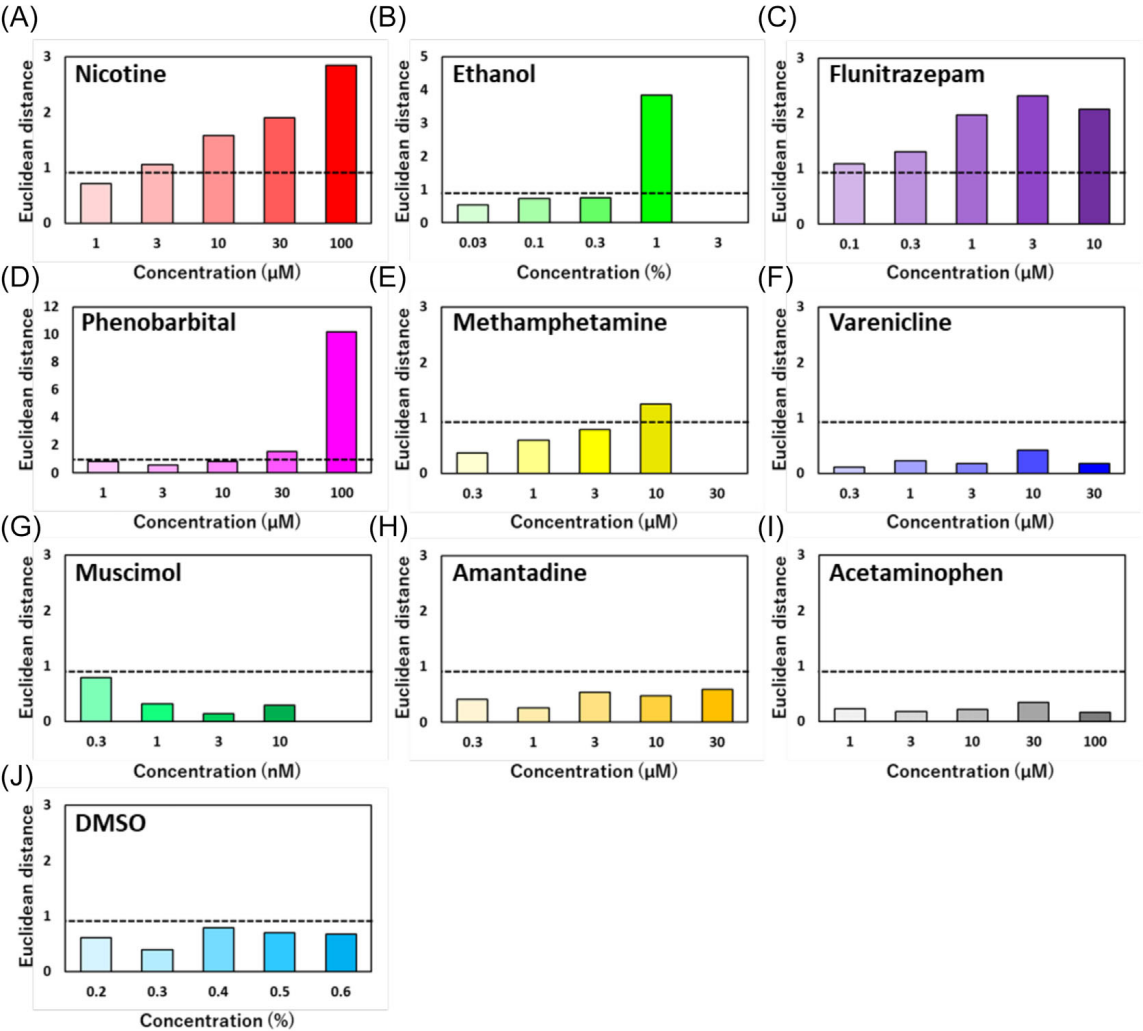
Distance on the two‐dimensional plane of PCA before and after chronic administration. As a criterion for changes before and after chronic administration, the threshold was set at 2SD of the distance before and after chronic administration of DMSO (dashed line). (A) Nicotine, (B) ethanol, (C) flunitrazepam, (D) phenobarbital, (E) methamphetamine, (F) varenicline, (G) muscimol, (H) amantadine, (I) acetaminophen and (J) DMSO.

**FIGURE 7:**
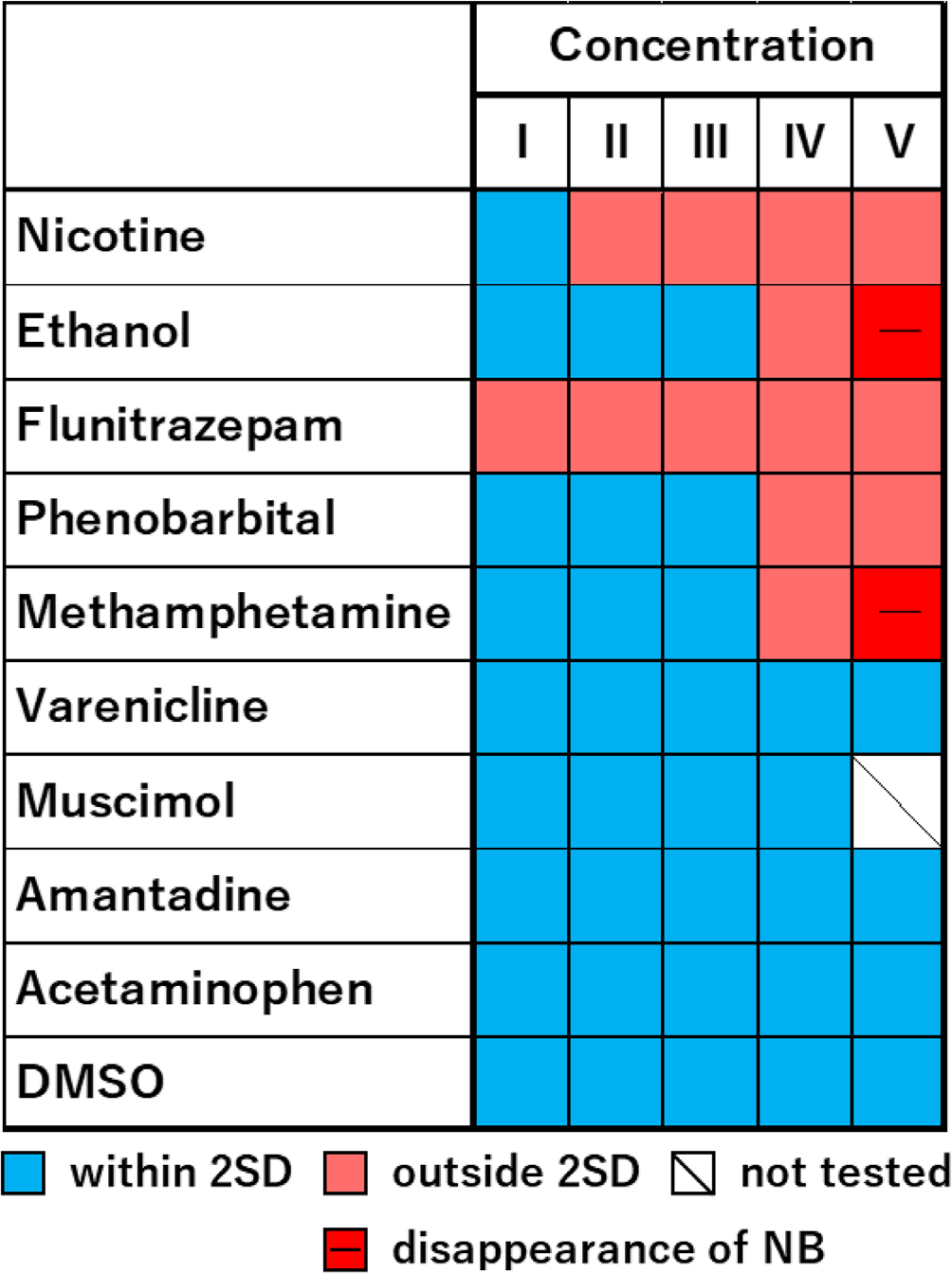
Detection of addictive compounds. The response that exceeded 2SD of the distance before and after chronic administration of DMSO was detected as an addictive‐like response. Nicotine (1, 3, 10, 30 and 100 μM), ethanol (0.03%, 0.1%, 0.3%, 1% and 3%), flunitrazepam (0.1, 0.3, 1, 3 and 10 μM), phenobarbital (1, 3, 10, 30 and 100 μM), methamphetamine (0.3, 1, 3, 10 and 30 μM), varenicline (0.3, 1, 3, 10 and 30 μM), muscimol (0.3, 1, 3, 10 and 30 nM), amantadine (0.3, 1, 3, 10 and 30 μM), acetaminophen (1, 3, 10, 30 and 100 μM) and DMSO (0.2%, 0.3%, 0.4%, 0.5% and 0.6%).

To detect specific changes in addictive compounds before and after chronic administration, we explored parameter sets where no significant differences were observed before and after chronic administration in non‐addictive compounds, while significant differences were observed only in addictive compounds. As a result, parameter sets including IBI, duration, IMFI and duration IQR were derived. The obtained parameter sets were then subjected to PCA. The results of PCA, plotted on a two‐dimensional plane using the first and second principal components, revealed distinct positions for addictive compounds before and after chronic administration (Figure 5). Additionally, the distance on the two‐dimensional plane, calculated as an indicator of changes before and after chronic administration, showed a concentration‐dependent increase in addictive compounds, while non‐addictive compounds did not exhibit a concentration‐dependent increase in distance (Figure 6). To establish criteria for changes before and after chronic administration, we set the threshold for the distance based on 2SD (twice the SD: 0.93) of the distance before and after chronic administration of DMSO. Attempting to detect addictive‐like responses, addictive compounds showed responses exceeding the threshold at specific concentrations, such as 3 μM or higher for nicotine, 1% or higher for ethanol, 0.1 μM or higher for flunitrazepam, 30 μM or higher for phenobarbital and 10 μM or higher for methamphetamine. Non‐addictive compounds did not exhibit addictive‐like responses under these conditions (Figure 7).

### 3.4 Changes in receptor expression levels before and after chronic administration

**FIGURE 8:**
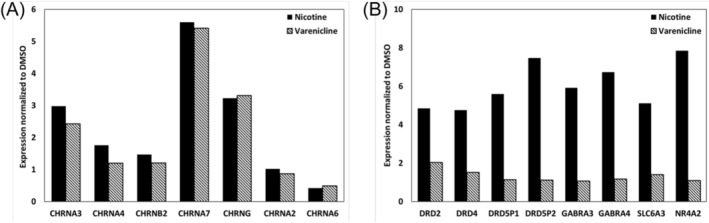
Changes in the expression levels of various receptors after chronic administration of nicotine and varenicline. Normalization of the expression levels of various receptors after chronic administration of DMSO, set to 1. (A) Changes in nicotine receptor expression. (B) Changes in dopamine receptor and GABAA receptor expression.

The results of the gene expression analysis of cultured samples after the test are presented (Figure 8). RNA from culture samples was extracted immediately after testing on Day 45. The expression levels of nicotine receptors were similar after nicotine and varenicline administration. However, the expression levels of dopamine receptors and GABA receptors were significantly higher with nicotine compared to varenicline. It became evident that chronic administration led to changes in the expression of various receptors, and a marked difference was observed in chronic administration of addictive compounds compared to non‐addictive compounds.

## 4 DISCUSSION

In order to detect specific changes before and after chronic exposure to addictive compounds, we derived a parameter set (IBI, duration, IMFI and duration IQR) where no significant differences were observed before and after chronic exposure to non‐addictive compounds, whereas significant differences were observed only with addictive compounds. For addictive compounds, all five tested substances demonstrated addictive‐like responses, and there were no false positives in identifying non‐addictive compounds as addictive, indicating the feasibility of this evaluation method in detecting addictive compounds. In a previous report, the evaluation of seizurogenic activity through neuronal MEA measurements identified TSs, CV of IMFI and duration IQR as effective parameter sets.^39^ In this study, only duration IQR coincided with the parameters identified previously. This suggests that the optimal parameter set for evaluating acute neurotoxicity differs from that for assessing chronic addiction. It highlights the importance of selecting parameters suitable for the specific neural function under evaluation.

In PCA, it is generally accepted to use dimensions where the cumulative contribution rate exceeds 80%. In Figure 5's PCA, four parameters were used, so calculations were actually performed from PC1 to PC4. The contribution rate for PC1 is 52.4% and for PC2 is 29.2%, making the cumulative contribution rate 81.6%, so using PC1 and PC2 in this study is considered appropriate. Because the high cumulative contribution rate was obtained using only the first two principal components, we deduced that the analytical parameter set derived in this study was important for the detection of addictive compounds.

In this study, five compounds were selected and used as addictive compounds. Nicotine, ethanol, flunitrazepam, phenobarbital and methamphetamine are compounds that have been the subject of numerous studies regarding their addictive properties. For non‐addictive compounds, we attempted to capture addiction‐related features that differ from the primary mechanism of action by selecting compounds with mechanisms of action similar to the selected addictive compounds as much as possible. By using PCA to evaluate changes in responsiveness before and after chronic administration, we successfully identified addictive versus non‐addictive compounds despite having similar mechanisms of action. The fact that compounds with similar mechanisms of action differ in terms of addiction suggests that factors such as receptor expression or neural network plasticity, separate from the primary mechanism of action, are influencing the outcome. The parameters used in PCA were IBI, duration, IMFI and duration IQR, suggesting that parameters related to the interval and duration of NBs reflect addiction‐related effects that differ from the primary mechanism of action.

Gene expression analysis of the cultured samples after the experiment suggested that nicotine may have higher expression levels of dopamine receptors and GABA receptors compared to varenicline (Figure 8B). Dopamine receptors, especially DRD2, are critical genes related to nicotine addiction.^40^ While reports indicate an increase in dopamine receptor expression due to nicotine in addicted patients, changes in dopamine receptor expression caused by varenicline have not been reported.^41^ In rat experiments, there were no significant changes in dopamine receptor expression after varenicline administration. Furthermore, after nicotine/varenicline administration, the expression levels of hippocampal GABAA receptors increased, but the change induced by varenicline was considerably smaller compared to nicotine.^42^ Additionally, nicotine receptors (CHRNA3, CHRNA4 and CHRNB2) have been reported to be associated with nicotine addiction.^43^ In rat experiments, nicotine/varenicline administration led to an increase in the expression levels of α3, α4 and β2 in brain tissue, with nicotine showing a more pronounced effect compared to varenicline.^44^ These trends in receptor expression changes observed in this study are similar to our findings and suggest the potential to evaluate addiction by mimicking in vivo conditions in cultured neurons. It suggests that changes in receptor expression after chronic compound administration may lead to changes in neuronal activity. This implies the possibility of interpreting addiction‐like responses in MEA measurements based on changes in receptor expression due to chronic compound administration.

In compound evaluations using MEA measurements, the impact of solvent addition can be influenced by the cell type.^30^, ^45^ In this study, changes in neuronal activity due to the addition of DMSO were observed in human iPS cell‐derived neurons (Figure 4). Therefore, to accurately assess the impact of compounds on neuronal activity, it is essential to consider the effects of solvent addition. In this evaluation method, the threshold for the impact of solvent addition on neuronal activity was set at 2SD of the changes induced by the solvent. In addiction assessments using this threshold, no significant concentration‐dependent changes or alterations before and after chronic administration were observed with DMSO, allowing for the evaluation of compounds without being affected by the solvent influence.

This study has several limitations. Pharmacological tests using MEA were conducted through cumulative administration. However, for compounds that may cause desensitization of neurotransmitter receptors or ion channels, it is necessary to avoid cumulative administration. In this study, there may be some impact on the response to compounds, but it was within an acceptable range to detect the effects of excessive neural activity induced by compounds. Cumulative administration has the advantage of improving throughput and reducing costs. However, it should be implemented considering the characteristics of the compounds to be evaluated. In the measurements before and after chronic administration, the compounds were cumulatively applied, and the highest concentration was set relatively high to obtain acute responses, raising concerns about the impact on the tests due to cytotoxicity and other factors. However, the cumulative administration test was repeated twice overall, and since the second test was conducted on the same culture sample after the first test, it indicates that there was no impact at the level of cell death. Additionally, even if there were acute neurotoxic effects at high concentrations, the evaluation of addiction‐like responses is not affected because the comparison of compounds is based on whether the response to the compound changes before and after chronic administration and the magnitude of those changes. Additionally, this study evaluated only a limited number of compounds. Future research should investigate a broader range of compound types.

In this study, we evaluated the changes in neuronal activity before and after 10 days of chronic administration. There is a concern that the changes in neuronal activity due to the maturation of neurons over the 10 days of chronic administration may be compounded with changes in the analysis parameters. However, the purpose of this study is to distinguish between addictive and non‐addictive compounds, and since the effects of neuronal maturation over the 10 days are equally present in all test conditions, the effects do not affect the comparison of acute responses before and after chronic administration of addictive and non‐addictive compounds. In the future, by advancing research comparing the presence or absence of chronic administration, it will be possible to accurately identify changes in neuronal activity due to addictive compounds.

In conclusion, MEA measurements of human iPS cell‐derived dopaminergic neurons demonstrated the detection of specific addiction‐like responses before and after chronic administration of addictive compounds. Furthermore, in tests conducted for reproducibility verification, precise discrimination between addictive and non‐addictive compounds was achievable using a similar evaluation method (Figure S1). However, depending on the type of addictive compound, the concentration at which addiction‐like responses were detected varied (Figure S2). This is presumed to be due to differences in the culture days of neuronal cells and the duration of chronic administration, highlighting the need for further investigation into the correlation between the expression of addiction‐like responses and culture days as well as chronic administration periods. The experiments for reproducibility verification were modified to start chronic administration from Days 30 to 40 of culture, and the duration of chronic administration was extended from 10 to 20 days. All other conditions remained the same. Additionally, conducting gene expression analysis on cultured samples after the tests revealed changes in the expression levels of various receptors due to chronic administration of addictive compounds, suggesting the potential interpretation of these expression changes as addiction‐like responses in MEA measurements. The addiction assessment method in human iPS dopamine neurons through MEA measurements conducted in this study proves to be effective in evaluating the addiction of compounds on the human nervous system and holds promise as one of the in vitro addiction testing methods, serving as an alternative to animal experiments.

## AUTHOR CONTRIBUTIONS

Yuto Ishibashi, Nami Nagafuku, Shingo Kimura and Ikuro Suzuki designed experiments. Nami Nagafuku and Shingo Kimura conducted experiments. Yuto Ishibashi and Xiaobo Han analysed the data. Yuto Ishibashi, Xiaobo Han and Ikuro Suzuki wrote the manuscript. Ikuro Suzuki supervised the project.

## CONFLICT OF INTEREST STATEMENT

The authors declare no conflict of interest.

## Supporting information


**Figure S1** **Distance on the two‐dimensional plane of PCA before and after chronic administration (reproducibility verification).** As a criterion for changes before and after chronic administration, the threshold was set at 2SD of the distance before and after chronic administration of DMSO (dashed line). (A) Nicotine, (B) Ethanol, (c) Flunitrazepam, (D) Phenobarbital, (E) Methamphetamine, (F) Varenicline, (G) Muscimol, (H) Amantadine, (I) Acetaminophen, (J) DMSO.

**Figure S2** **Figure 6 Detection of addictive compounds (reproducibility verification).** The response that exceeded 2SD of the distance before and after chronic administration of DMSO was detected as an addictive‐like response. Nicotine (1, 3, 10, 30, 100 μM), ethanol (0.03, 0.1, 0.3, 1, 3%), flunitrazepam (0.1, 0.3, 1, 3, 10 μM), phenobarbital (1, 3, 10, 30, 100 μM), methamphetamine (0.3, 1, 3, 10, 30 μM), varenicline (0.3, 1, 3, 10, 30 μM), muscimol (0.3, 1, 3, 10, 30 nM), Amantadine (0.3, 1, 3, 10, 30 μM), Acetaminophen (1, 3, 10, 30, 100 μM), DMSO (0.2, 0.3, 0.4, 0.5, 0.6%).

## Data Availability

The data that support the findings of this study are available from the corresponding author upon reasonable request.
