# Supplementary figures and images for: Development of an evaluation method for addictive compounds based on electrical activity of human iPS cell‐derived dopaminergic neurons using microelectrode array
Source: Addict Biol. 2024 Oct 9;29(10):e13443. doi: 10.1111/adb.13443 (PMC11462589; doi:10.1111/adb.13443)

**A**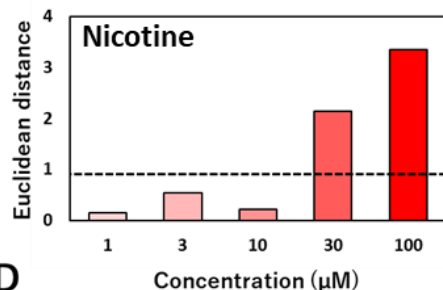**B**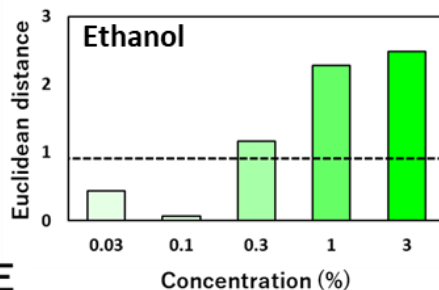**C**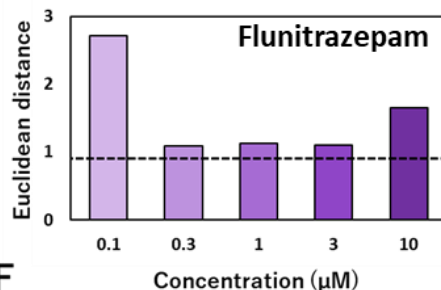**D**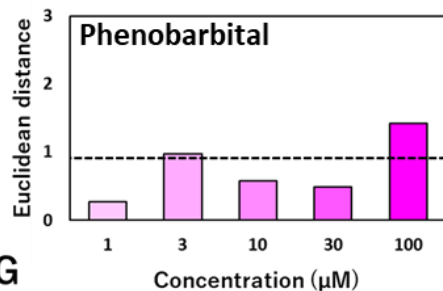**E**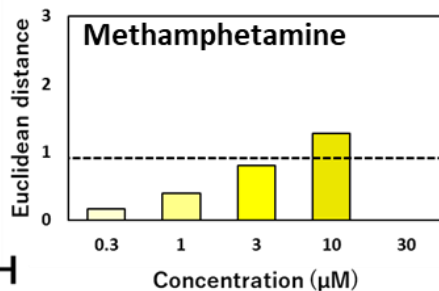**F**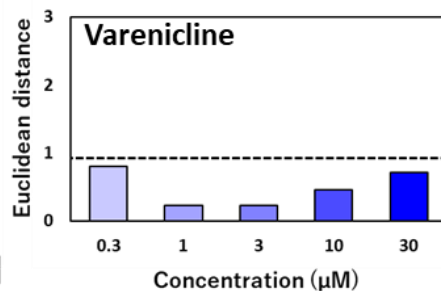**G**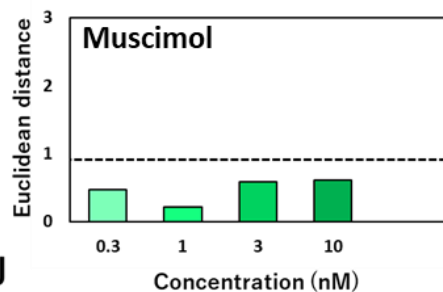**H**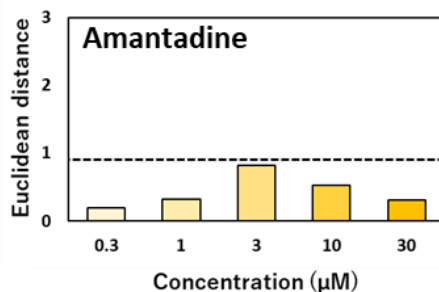**I**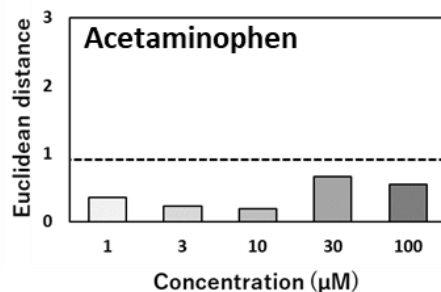**J**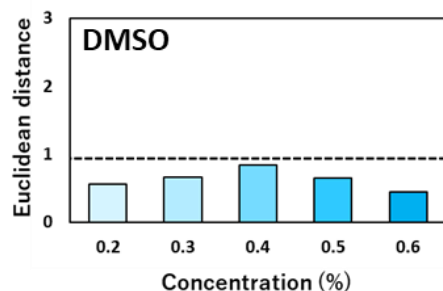

Supplement: Supplementary file 1 — Figure S1 Distance on the two‐dimensional plane of PCA before and after chronic administration (reproducibility verification). As a criterion for changes before and after chronic administration, the threshold was set at 2SD of the distance before and after chronic administration of DMSO (dashed line). (A) Nicotine, (B) Ethanol, (c) Flunitrazepam, (D) Phenobarbital, (E) Methamphetamine, (F) Varenicline, (G) Muscimol, (H) Amantadine, (I) Acetaminophen, (J) DMSO. [file ADB-29-e13443-s001.pdf]
