# Supplementary material for: Development of an evaluation method for addictive compounds based on electrical activity of human iPS cell‐derived dopaminergic neurons using microelectrode array
Source: Addict Biol. 2024 Oct 9;29(10):e13443. doi: 10.1111/adb.13443 (PMC11462589; doi:10.1111/adb.13443)
Supplement: Supplementary file 2 — Figure S2 Figure 6 Detection of addictive compounds (reproducibility verification). The response that exceeded 2SD of the distance before and after chronic administration of DMSO was detected as an addictive‐like response. Nicotine (1, 3, 10, 30, 100 μM), ethanol (0.03, 0.1, 0.3, 1, 3%), flunitrazepam (0.1, 0.3, 1, 3, 10 μM), phenobarbital (1, 3, 10, 30, 100 μM), methamphetamine (0.3, 1, 3, 10, 30 μM), varenicline (0.3, 1, 3, 10, 30 μM), muscimol (0.3, 1, 3, 10, 30 nM), Amantadine (0.3, 1, 3, 10, 30 μM), Acetaminophen (1, 3, 10, 30, 100 μM), DMSO (0.2, 0.3, 0.4, 0.5, 0.6%). [file ADB-29-e13443-s002.pdf]

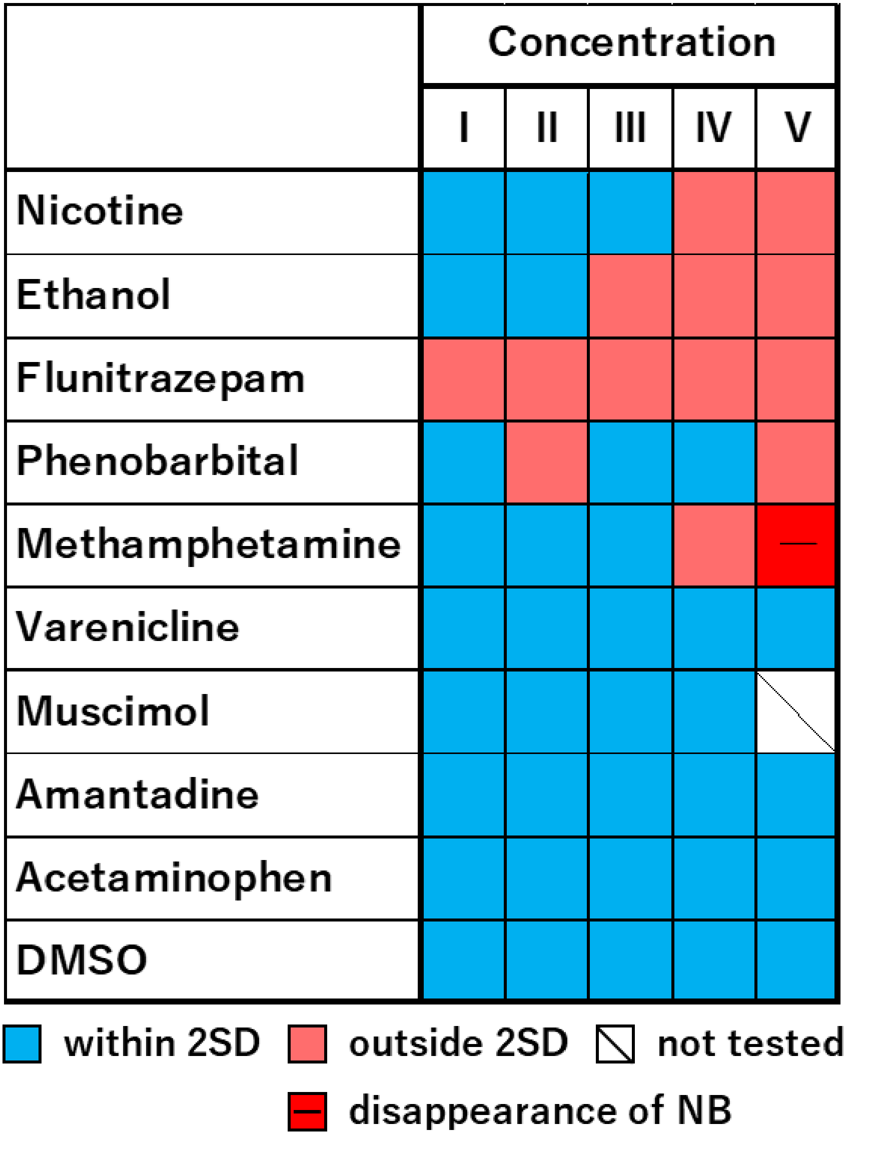
Concentration
I
II
III
IV
V
Nicotine
Ethanol
Flunitrazepam
Phenobarbital
Methamphetamine
Varenicline
Muscimol
Amantadine
Acetaminophen
DMSO
within 2SD
outside 2SD
not tested
disappearance of NB
